# Collectanea Medica, Consisting of Anecdotes, Facts, Extracts, Illustrations, Queries, Suggestions, &c.

**Published:** 1817-10

**Authors:** 


### COLLECTANEA MEDICA,

CONSISTING OF

### ANECDOTES, FACTS, EXTRACTS, ILLUSTRATIONS, QUERIES, SUGGESTIONS, &c.

RELATING TO THE

*History or the Art of Medicine, and the Auxiliary Sciences.*

---

Quicquid agunt medici, nostri farrago libelli.

---

THE memoirs of Madame de Stael, during the period of her more active life, have been detailed in so many different Journals, that we can scarcely suppose they have not fallen in the way of most of our readers. We had, however, prepared an epitome of the more striking events relating to her, and intended it as a part of a Biographical Collectanea: but, finding no readier means of explaining ourselves on one article in nosology than by offering the extract succeeding this communication, we have deferred our other Memoirs of dead and living Physicians till our next Number.

*Account of the long Illness of Madame de Stael, her Death, and Appearances on a subsequent Examination;* by M. PORTAL, her family Physician.

The Baroness de Stael, daughter of the celebrated M. Necker, the last Minister of Finance to the unfortunate Louis XVI. was equally celebrated by her highly-esteemed writings and her political opinions, which had caused her to be exiled from France during the Revolution, and gained her a flattering reception by the principal princes of Europe.

Madame de Stael, whose physician I had been from her infancy, as well as that of her father, consulted me, on her return to Paris, for an œdematous swelling in her legs, with which she had been afflicted some time, and which gradually became worse. Her complexion, naturally dark, grew still darker; and her eyes even assumed a yellow colour. Her digestion was painful. She experienced great restlessness and want of sleep, which she had long been unable to relieve by the use of one or two grains of opium, which she had taken every evening. Although about 53, she had only recently ceased to menstruate.

I thought it necessary to prescribe aperitives, slightly diuretic pills, with medicinal soap; extracts of saponaria officinalis, hops, and gentian, in equal quantities, mixed with bullock's gall: four of these pills, of four grains each, were given in the morning, fasting, at twice, an hour elapsing between them; and two cups of a tisanne made with the roots of the *rumex acutus* and *triticum repens*, with the the leaves of scolopendra, in which was infused a pinch of chervil, and to which were added ten grains of salt of nitre.

This simple treatment, in a few days, restored the urine, and diminished the œdema. Having, however, experienced light alvine evacuations, and recollecting that she had some time before been troubled with a looseness, against which several tonics had been prescribed with effect, with a little more opium than she was in the habit of taking, in the evening she thought proper to consult another physician, who prescribed her very irritating powders, which soon made the alvine evacuations cease. Madame de Stael profited by this interval to make visits in Paris, and receive much society at her own house; but the œdematous state of the legs becoming even worse than before, and her complexion yellower, I was again consulted, and prescribed the same treatment as before, in order to restore the urine and stools. My prescription had a prompt success; but the patient was frightened at some slight bilious evacuations, though necessary, which the medicines produced, and she again consulted another physician, who advised her to take a larger dose of opium than usual. Costiveness ensued, the urine was considerably diminished, the skin became of a darker yellow than ever, and Madame de Stael fell into a profound stupor, which lasted so long as to create great alarm. I was once more consulted, and found in the pulse a decided fever: the urine was in a very small quantity, and very red, leaving a sediment still more so; the tongue was red; the lips and cheeks of the same colour; the rest of the face very yellow, and puffed; the hands and feet particularly œdematous. I prescribed strong lemonade, and in each glass a few drops of nitrous æther. *Lavements*, slightly purgative, were given. She recovered from the stupor, but the fever was really commenced, and became much worse in the evening. The urine was still in small quantity, red, and thick.

It appeared to me to be a real bilious fever, inasmuch as by the feel a swelling was discovered on the right hypochondrium. I was persuaded that the treatment ought to consist in mild medicines (the irritation being extreme) with aperients. The patient diminished the quantity of lemonade, to take, from time to time, chicken broth, with nitre, and a beverage of an infusion of triticum repens (dog's grass) and chervil, also with nitre, sweetened with the syrup of the *five aperitive roots.* This treatment was seconded by emollient clysters, and the patient was evidently much better. On the sixth or seventh day, I advised her drinking the waters of Vichy, at first mixed with chicken-broth, and afterwards pure, and at the end of the sickness to add acetate of potass.

This treatment completely succeeded, as the fever diminished daily, and ended about the fourteenth day. The urine gradually became more abundant and clear, and the alvine excretions had proportionally become yellow and bilious, instead of grey, as before. The puffing-up of the face, and the œdematous state of the hands and feet, were considerably diminished. The region of the liver was

2 neither neither so protuberant nor so hard: that of the spleen, however, remained a little tumefied.

It was towards the decline of this bilious fever that a skilful physician, M. Lucas, was called in. He thought it proper to continue the treatment I had prescribed, and confirmed my prescription of the waters of Vichy, united with the acetate of potass. He thought the moment favourable for it. At first half a drachm only was prescribed for the two glasses the patient drank in the morning, and by degrees the dose was augmented to a drachm in each glass. The alvine evacuations continued bilious without being abundant.

Madame de Stael appeared to get better every day. She was advised to get up, but, having felt some difficulty in standing, and more so in walking, she was soon obliged to remain in bed. She long complained of pain and spasms in the lower extremities; the urine became less; the skin resumed its yellow colour; she had borborigmi, with a tumid abdomen, but not hard; the œdema of the lower extremities increased; that of the hands and even the arms was considerable.

Known diuretics were prescribed, as the tisane (beverage) of the five aperient roots, in which was infused chervil, and to which was added oxymel of squills. Slightly purgative clysters were also recommended, yet the œdema, or rather the anasarca, which increased, appeared to point out the proper use of blisters to the legs, which we were the more readily inclined to, as the patient had had, for several years, an eruption in the face, but which had long disappeared, yet we thought it necessary to take it into consideration.

The waters of Vichy were suspended, and replaced by the juices of plants, as water-cresses, borage *(bourroche)*, dandelion, chervil, water trefoil, and live millepedes, bruised, in large quantities. These juices, well purified, with the addition of oxymel of squills, were given in the morning, in the dose of five or six ounces at twice. The patient also took, in the course of the day, a few glasses of an infusion of hops and chervil, with nitrous æther and tincture of digitalis, which was also employed in frictions, in powder, in a mucilaginous liquor.

This treatment was so efficacious that Mad. de S. was enabled to go out in a carriage, as the weather was fine; but this recovery did not continue. She complained one day, in returning from her drive, that, in getting into her barouche, she had bruised one of her feet; that the pains increased, and she could not go a single step or support herself on her legs, feeling a stiffness and extreme weakness in the upper extremities, and more so in the lower ones, though at intervals they were very painful.

A skilful physician, called in, thinking the seat of the disorder in the liver, advised mercury in a gummy excipient, to which I could not subscribe, not thinking the seat of the disorder confined to that part, and mercury not appearing to me to be proper in the case, especially as there were ulcers on the tongue and in the mouth. This treatment was, however, tried, but soon abandoned, as the patient became much worse.

I wished to examine the state of the matrix. A celebrated accoucheur, who was called in, thought it was enlarged. This was contradicted by an able surgeon, who decided that this organ was in no way altered; but he fancied the disorder lay in the spinal marrow, and that there was an extravasation in the vertebral canal. He thought blisters applied along the spine would be serviceable, and recommended a tonic liniment for the extremities, and even on the spine: this last article was exactly followed.

Numerous consultations followed, in which similar remedies were prescribed without success. A friction of tincture of phosphorus in like manner failed. The difficulty of motion in the lower extremities became greater; the upper ones, however, were a little more flexible. Mad. de S. complained of a pressure in the upper part of the breast, on which a physician, newly called in, applied a blister. Another well-known physician fancied he discovered symptoms of hydrothorax, and that he even heard a kind of undulation in the cavity, by means of a sheet of paper rolled up like a trumpet, and applied to the breast and his ear. I certainly did not agree in his opinion, or method of proof, as the swellings had decreased, the urine re-established, and she was able to lie in her bed horizontally all day; but, as the spasms continued, the same physician thought adviseable to apply two plates of magnetised steel to the breast: the inefficacy of this was soon proved.

Mad. de S. became very thin: the stiffness continued, and she could not walk. Exciting and cooling remedies were applied with the same effect.

A physician of Geneva was joined to those of Paris: he proposed the internal use of mustard, to restore the nervous systems, and stimulating ointments on the spine, together with an infusion of bark that I had prescribed for some time. But there was already an impression of gangrene in the region of the os coccygis, and two or three spots of that nature on the left lower extremity, which determined us on proposing strong doses of the antiseptic remedy, so often successful; but the progress of the gangrene was so rapid that all the succours of art were unavailing. Madame de Stael died the 14th July, at four o'clock in the morning, after an illness of upwards of four months.

Her body was opened and embalmed. I was not called to attend this operation, but I learnt from M. Jurine, who was present, that there was neither dropsy in the chest nor any disorder in the spinal marrow or the vertebral canal, no extravasation, &c. The viscera were in a good state; the liver only appeared hardened in some parts longitudinally, but nothing to cause so severe an illness. I think my treatment had produced good effects.

May not some reproaches be made on the abuse of this treatment, and the employment of contrary remedies? As the ordinary physician knew the patient well, and her disorder, and the success he had already obtained, ought it not to have inspired some confidence, nay entire confidence, as well in the patient as her family? Madame de S. was evidently afflicted with cachexy, which the bilious lious fever augmented; and was this even to a certainty removed? It is often followed by dropsy, and sometimes stupor and paralysis even of the lower extremities.

Would not the continuation of the juice of scorbutic and diuretic plants, which I had began to prescribe with success, to which was added the use of known depuratives, have been preferable to so many remedies which were administered, as the patient had been stationary for weeks, notwithstanding the use of stimulants prescribed on the one hand, and on the other cooling opiates. Extract of henbane was at one time proposed.

May not such treatment, continued for several months, have produced, or at least concurred, to the gangrene which was so rapidly followed by the death of Madame de Stael? PORTAL.

We have inserted the above, not with any view to canvas the practice of the Parisian physicians, but as a specimen of the pharmacy of that city, of which we shall hereafter make further mention.

---

### *Selections on the Elephantiasis of Aretæus.*

THE Elephantiasis of Aretæus, or Lepra Arabum, has lately occurred in the Hospital at Edinburgh, a part of the world in which it was unknown in the days of the illustrious Professor Cullen. The suspicions of its infectious property, and of the salacity of the unhappy patients, was at one time so great, that Dr. Bateman, in a paper in Dr. Rees's Cyclopœdia, did not scruple to consider the transmission of the disease from Africa to the West-India islands as a just punishment to the Creoles for the detestable practice of dealing in human flesh. Though, in his Synopsis, he has entirely retracted this opinion, yet he still seems to consider a salacious character as a part of the disease. As the subject will occur in our notice of Mr. Good's Nosology, in this Number, we have thought it might be desirable to bring the facts, and the authorities on which they rest, before our readers.

Dr. Heberden, to whose ardour and industry in promoting medical knowledge we owe the first suggestion of the "Medical Transactions" of the London College, having a brother in Madeira, was not likely to lose such an opportunity of learning the history of a disease unknown in the northern regions. That gentleman's remark is, "Notwithstanding the just abhorrence of this loathsome disease, it certainly is not so contagious as is generally imagined, for I have never heard of any one who has contracted the distemper by contact of a leper; and, on the contrary, am a constant witness of communication between lepers and other people without the least ill consequence." He afterwards admits the hereditary property of the disease.

Dr. Adams, from the same island, comes next in order. As his account is official, and also as he is the first to mention that the hereditary extension of so loathsome and incurable a disease is checked by a provision in the progress of the disease itself, we shall transcribe his account more at length.

*Memorial presented to His Excellency Don Jose da Camera e Leme, concerning the Inhabitants of the Lazaretto near Funchall.* By JOSEPH ADAMS, M.D. Extra Licentiate of the Royal College of Physicians in London, and Physician in the Island of Madeira. With a further Account of that Institution.

The unfortunate objects of the lazaretto, near Funchall, cannot but attract the notice of every stranger: a disease entirely unknown in the colder regions, must, in a particular manner, excite the attention of an English physician, were it only from observing that of some of the men, whose scalp is well covered with hair, the chin is perfectly smooth. I had also remarked, that this want of beard is in some attended with a delicacy of voice, not indeed such as we perceive in eunuchs, but rather like that of boys. Still a doubt remained whether this might arise from any change in the figure of the throat, which in most is considerably deformed by irregular prominencies. The voices of some are also very nasal, from similar causes.

The first person we examined was a youth named Gonzalves, about twenty-three years of age. His face is strongly marked with the disease, with which he has been several years afflicted, though he has resided only two years in the house. His voice is nasal, his uvula either lost or concealed by the tubercles about his palate. Has no hair on his chin or pubes; his testicles can scarcely be felt; his scrotum, and all the organs, very much resemble those of a boy of six or seven years old, excepting that the prepuce is somewhat elongated. With all this he is cheerful, being free from pain; and, when asked whether the state of his throat made it difficult for him to swallow, answered, that his only difficulty was in procuring enough to swallow. His father died of the disease.

The second, who passed under examination, was said to be one of fifteen children, born of both healthy parents, and grand parents. Of these children, four died young, and, by his account, of the disease, the rest are all healthy. The chin and genitals in this youth are as in the former subject, though he is considerably advanced beyond the age of puberty.

Manoel Fernandez is thirty-six years old; has been ill thirteen years; says he never had a beard; has but little hair on the pubes. His testicles are much wasted; has only one eye.

The above cases are sufficient to show, what was afterwards proved by an accurate general examination, that, when the disease attacks a male subject before the age of puberty, he never acquires that state; and that such as are affected later in life, gradually lose the power of procreation, as far as can be judged by the changes which take place in their organs.

The proofs of a defective organization in the women are scarcely less striking, as far as those organs can be examined.

Chronic

Chronic diseases, which seem to arise spontaneously, and which never yield to the unassisted powers of the constitution, must have their cause in climate, predisposition in the patient's constitution, peculiarity of diet, or probably in all. If the first and third causes only exist, we may hope for relief in change of climate and mode of living; but if the disease never occurs, excepting where there is an original predisposition, the cure can only be permanent as long as the patient is removed from the exciting causes. As the predisposition is born with the patient, the inference follows, that he must have derived it from his parents; we are, therefore, apt hastily to conclude, that his offspring must be infected also; and because this sometimes occur, we fix no limits to our extravagant terrors concerning what are called hereditary diseases. We do not consider, that, unless the disease can be traced back to our first parent, and thus involve us all, it must have originated in one whose ancestors, from the beginning of the world, were free from it. Still less do we take notice, that, when the disease has appeared in a family, it often ceases with the individual who is the first subject of it, and can rarely be traced beyond his immediate offspring.

It is not my intention to dispute that the predisposition to leprosy is hereditary. The number of subjects in the lazar house, from whose parents the disease may be traced, is sufficient to establish this fact. It is, however, a most important remark, that all those subjects, who appear to derive the disease from their parents, have been attacked at an early age; and, as far as we could learn, in those in whom the disease has shown itself at a later period, it has originated with themselves. It is not less notorious, that the disease has gradually disappeared among the wealthier families, since they have adopted a more generous diet, and since the increased mildness of the government has relieved them from anxieties, as destructive to health as poverty or want. The disease, though at present nearly confined to those whose diet is the poorest and most precarious, is yet, in a few instances, found to visit such whose situation is above want: and many, in the most abject state of poverty, escape it. Hence it follows, that no external cause will produce it, unless the constitution is predisposed to it. Now a predisposition must be derived from the parents, whether they are diseased or not.

Having thus, as far as it can be, settled the question concerning the hereditary nature of the disease, our next enquiry should be, how far it is infectious; but, as this more immediately belongs to the mode of treatment, I shall first proceed to mark the particular signs by which the disease may be ascertained.

Among the unequivocal marks, we may consider tubercles about the face, particularly on the external ear, *alæ nasi*, eye-brows, or forehead. These tubercles, till an advanced stage of the disease, are not only smooth, but have, for the most part, a higher complexion than the natural skin, approaching nearer to the sanguineous hue, appearing as if semi-transparent, splendid as if the surface were smeared with oil, and, on a closer examination, sometimes exhibiting small blood-vessels ramifying on their surface. This appearance is not equally marked in all; but the following may serve as their general history and form.

[This description is longer than our limits will permit, and, in the face, not materially different from other authors.]

The body usually escapes better than the limbs, but is not always entirely free. The genitals have been already described.

In the upper and anterior part of the thigh, nearly in contact with the lower part of the scrotum, there is, in almost every case, a firm (to appearance) glandular swelling, moveable and prominent, or concealed, according as the patient is fat or lean, or in proportion to the progress of the disease. It is remarkable that none of the women are without it. In most of the men these tumours are particularly prominent, extending gradually upwards. In some there are also inguinal buboes. In every case the swellings are indolent, never giving pain, nor becoming discoloured, nor showing any disposition to suppurate.

When the disease arrives at such a state, as to distort the lip, nose, eye-brow, or lobes of the ear, it will require little more than common observation to detect it. But, if these are so little affected as to render it doubtful, we should always examine the upper anterior part of the thigh, towards the genitals, for that tumour, which I have called glandular, but the exact nature of which cannot be ascertained without dissection. In the mean time it may be easily distinguished from those buboes in the groin, which are formed by venereal or any other causes, producing inflammation in the genitals or lower extremities. These are usually in the hollow of the groin, hard, and immoveable till they begin to lessen, as inflammation subsides. On the contrary, the tumour from leprosy is never very hard, always more or less moveable, without pain or discolouration of the integuments, usually much larger than the common bubo, and lower in its situation. There is, indeed, some variety in the height, size, and mobility, but never sufficient to confound it with the inguinal bubo. It should be further remarked, that in a few instances I have found the inguinal bubo and femoral tumour in the same subject. In two subjects also, in whom the disease was, in other respects, well marked, I have discovered only a general thickening of the integuments over the part where the femoral tumour is usually found. In one of these was a similar, though smaller, tumour in the hand. In young subjects, particularly boys, before the age of puberty, if we see any marks of approach towards that state, we ought to suspend our judgment, till we discover signs of the disease the most unequivocal and certain.

The two diseases, with which that of the lazar house has been confounded, are the leprosy of the Greeks, and the elephantiasis of the moderns, or, as it is usually termed in England, the Barbadoes leg. This confusion has been much increased, because the disease of the lazar house has been by some authors called leprosy, and by others elephantiasis, or even in the same work has been called by both names. It is true, that the most accurate among the ancient writers use no other term than elephantiasis: but that this confusion has existed among the moderns, will appear by quotations from two well received authors, whose object was to characterize the diseases with more accuracy than their predecessors. Dr. Mead remarks, that "the Syrian leprosy," [which before he calls elephantiasis], "did not differ in nature, but degree, from the leprosy of the Greeks;" and Vogel, after describing the elephantiasis of the ancients, adds, as a division elephantia; "Eadem duntaxat in pede valde tumido et duro." Thus we see Mead considers leprosy and elephantiasis as the same disease, without hinting at the enlarged limb; and Vogel considers the elephantiasis of the ancients and the moderns as the same disease, only attacking different parts. It may, therefore, be right to observe, that when I use the single term elephantiasis, my wish is to confine it to the modern disease.

Thus we have,

1. The Arabian or Syrian leprosy, or elephantiasis of the ancients; the disease already described.

2. The leprosy of the Greeks.

3. The elephantiasis, or elephantiasis of the moderns, called also the Barbadoes leg.

[A description of the two latter diseases follows.]

To conclude this part of the subject, it may be enough to remark generally:

1st. That lepra Grecorum is distinguishable by the scales being coeval with, and as general as the disease—whilst in lepra arabum they never appear but in an advanced stage, and then only partially.

2dly. That elephantiasis of the moderns, or the Barbadoes leg, is distinguishable by the enlarged glands being always in the groin —by the pain and increase they suffer with each returning paroxysm of fever—by the sudden swelling of the limb at the same time—by the thickening of the skin, without any loose furfuraceous scales, and by the tubercles, if there are any, being horny and rough, instead of the semi-transparent smoothness of those in the Arabian leprosy.

Though the lazars rarely arrive at old age, they, for the most part, seem to die of other complaints. I recollect, at the period of an influenza, these people suffered earlier and more severely than any others.

Though, by the account from a woman who resided for some time in the house, it is certain that the disease cannot be highly infectious, yet it is right to remark some circumstances, which I shall leave others to determine, whether they should be considered as merely coincident.

The porter of the house is become a lazar since his residence in the lazaretto: but he is the only servant, since its establishment, who has shown the disease.

Besides the married couple now in the house, the accounts I collected mention two other women, whose husbands were lazars; but in these cases we must recollect the same mode of life, local residence, and probably some consanguinity.

2 It

It appears not only that the Greek writers were unacquainted with the swelled leg and foot, which afterwards acquired the name of elephantiasis among the Arabs, but that the Latins were ignorant both of this and of the elephantiasis of the Greeks. By an attention to these particulars, we shall readily discover the cause of that confusion of names above mentioned.

When the medical school was transferred from Greece to Arabia, where the swelled leg and foot was a frequent disease, and the elephant often in sight, it was impossible not to be struck with the similarity. I pretend not to determine whether the Arabs had previously distinguished this swelling by a name which had reference to that animal; but it is certain that the term elephantiasis was by them applied to a disease no where noticed by the Greek physicians, from whom the word was adopted.

When the sciences found their way back to the west, and the Salernian school was established, it is well known that the Arabic writers were at first the principal sources of information: and, as the Arabians had not only appropriated the term elephantiasis to a disease of their own, but had given the term leprosy to elephantiasis of the Greeks, a confusion must have arisen in the translations through the different languages. However, the Latins soon found it necessary to distinguish between a disease which was easily cured, viz. the leprosy of the Greeks, and one which the old writers had denominated a cancer of the whole body; that is, a disease gradually increasing in whatever part it commenced, and without a remedy. Hence the distinction between *lepra Grecorum* and *lepra Arabum*, which last they perceived must be the elephantiasis of the Greeks.

Though they had thus discovered the difference between the Greek and Arabian leprosy, they had less inducement to inquire after the Greek and Arabian elephantiasis, because they were equally ignorant of both, or saw them so seldom, as to confound them with the more intractable state of other diseases, with which they were better acquainted. At length the appearance of syphilis produced a more accurate research whether this disease could be discovered in the writings of the fathers of physic who, about this time, began to be studied in their native tongues. It was then found that the Arabian writers confined the term elephantiasis to a disease in which the leg and foot are much swelled, and which was unnoticed by the Greeks.

To pursue the subject through the whole controversy, would be more ostentatious than useful. Those who wish to follow it beyond the quotations produced by Lorry, may consult Sebastian Aquilianus, Nicol. Leonicenus, and several others, preserved in the Luisinian collection. To do justice to the industry of these writers, we should recollect, that the works of Aretæus were not recovered from the rubbish under which they were concealed for near half a century after that time. The Italians, therefore, could only discover the meaning of the Asiatic Greeks from the writings of Galen and his commentators, whose descriptions are very obscure, and scattered in different parts of his works.—*Adams on Morbid Poisons*, second edition, 1807.

Dr.

Dr. Gourlay, in his History of Madeira, considers this wasting of the testicles as a leading character of the disease.

About six years ago, a case occurred at St. Bartholomew's Hospital. The subject was a mulatto, and, though by no means young, had no hair on the pubes. He died in the house, and his body was afterwards examined. We do not recollect any record of the event, so that, if any gentleman, a student at the time, has preserved minutes, we shall very thankfully receive them.

The next case was in the same house, and particularly interesting, by having attracted the notice of a considerable number of the faculty in the metropolis, and by being recorded in several collections. The following is Mr. Lawrence's account in the sixth volume of the Medico-Chirurg. Trans.

"An inguinal gland on each side was rather more distinct than usual. The condition of the generative organs corresponded with the description of Dr. Adams just alluded to. Not only had their development been arrested from the time when the disease broke out, but they had actually undergone diminution and decay. The scrotum was shrivelled, and seemed empty; the testes could with difficulty be felt; they were soft, and about the size of small horse-beans."

The following is Dr. Roberts's account of the same subject in the Med. Trans. of the London College of Physicians:—

"His genitals diminished in size, from which also the hair disappeared, as well as from the eye-brows and arm-pits; that, after his landing, some tubercles were formed also on the hands and feet, and he confessed, what might reasonably be expected, that he had no sexual desires:"—"there were, besides, glandular tumours near the groin on both sides."

The last case is contained in the Medico-Chirurg. Journal, and was transcribed in our last Retrospect.

We conceive from this time it will be impossible to mistake a disease so strongly marked, and, whilst we must feel every sentiment of commiseration for sufferers so wretched that nature seems on that account to have placed a *veto* against their propagation, it is at least some relief to reflect that they are no longer to be dreaded as formidable to the softer sex, or dangerous by an intercourse with the rest of the world. Under these apprehensions, they were once driven into wilds and uninhabited deserts: in the progress of improved society, they were only sentenced to a solitary confinement: in the present improved state of knowledge, they are permitted all the few enjoyments their unhappy condition permits.

---

### *Account of the fatal Accident which happened in the Leadhills Company's Mines, the 1st March, 1817.* By Mr. James Braid, Surgeon, Leadhills.

On 1st March last, I was sent for, about seven o'clock A. M., to try if any thing could be done for a number of men, who were found found in the Leadhills Company's mines, who appeared to be suffocated.

On the 30th December, 1816, a young man, who kept a fire-engine nearly 600 feet below the surface, was found dead, and the air where he was not, to be at all agreeable:—the usual modes of resuscitation were tried, but without any good effects.

On the 24th of February, 1817, there were several men very severely affected from the bad state of the air, but, by giving them gentle laxatives, and keeping them quiet, they got pretty well again in the course of a few days.

On the 1st of March, none of the men had got to bank when I arrived, except a few who had been down only a very short time, and returned upon finding the air so bad. By and bye, a number of those who had been down for a short time, at 25 fathoms, were brought up, and most of them quite furious. Some were disposed to fight; others, supposing every one they saw disposed to lay hands on them, made efforts, under the most extreme terror, to escape; others, quite listless, appeared to take no notice of what was going on around them. Some were singing, and some praying. Many were as if intoxicated with ardent spirits: those who had seen them in that state assured me their actions were very much the same.

Many of them vomited, and others had the inclination, but could not do so. Some evacuated the contents of the rectum, and others had the desire without effect. The pulse was different—in some remarkably quick and feeble; in others slow, feeble, and irregular. Most complained of insufferable head-ache, which was somewhat relieved after vomiting. To those who had a desire to vomit without effect, I gave an emetic of sulphate of zinc; and to those troubled with tenesmus, a laxative glyster: both were followed by an alteration of symptoms.

In the course of two or three hours from the time they were brought to bank, the pulse was greatly accelerated, and hard. I prescribed a brisk purgative, after the operation of which they found themselves greatly relieved, and, by enjoining a cooling regimen, most of them got pretty well, in the course of a few days, without any other medicines. Upon inquiring of the men how it affected them, they said they first felt a difficulty of breathing, and had frequent involuntary deep inspirations—then a violent pain and beating in the head, with ringing of the ears—the inferior extremities became weak, and very painful immediately above the knees, and they could with difficulty support the body—the heart palpitated violently—great anxiety, and in some followed by vomiting. They now became giddy, and lost all recollection, and were, as has been remarked, affected as if they had taken a large dose of ardent spirits.

There were four men, however, at 25 fathoms, who were irrecoverably lost through their own imprudence of going to work at irregular hours. Though six o'clock A. M. was the proper hour, two had gone before four, and other two a little after, in order that that they might get out so much sooner. Such practices are not sanctioned by the masters. When they came to the bad air, they had thought to force their way through it, expecting it to be better below; but it had soon produced its deleterious effects upon them, so as to make them unable, either to go further, or to retrace their steps; and then, unable to support themselves, they had fallen, and remained amongst the bad air till assistance came too late. Animation must have been gone two or three hours before they were brought to bank, for they had been down not less than four hours. Had they not gone till the regular shift, when the air was found to be so bad, they would not have proceeded so far; and, if one or two had fallen, the others would have found some means to have rescued them, before they had been irrecoverably gone. It is presumed, the accident happened from a quantity of smoke escaping from the chimney of the engine under ground, into the way-gates, about the twenty-five fathoms, and so contaminating the air in the workings, from the quantity of sulphurous acid gas which the smoke contained, such as to render it unfit to support animal life, or rather, highly deleterious. The men described the air to be the same as where sulphur is burning slowly, and, consequently, sulphurous acid gas forming.

At the time the accident happened, the atmosphere was foggy, and there was a want of a proper current of air in the workings, and, in consequence of the stagnation, the air in that part of the way-gates, where the smoke was escaping, became so contaminated, by sulphurous acid gas, as to render it highly deleterious to animal life. A trap-door being opened, in order to save those who were still alive, about the twenty-five fathoms, (that is, those who went at the time for a regular shift, the four who had gone in before were dead when these men went to them,) and to enable others to give them assistance, with safety to themselves, the bad air immediately rushed down to the lower workings, and began to exert its deleterious effects upon those at eighty fathoms, who were effecting their escape by another shaft. All who were any length of time in this situation were violently affected in the manner already mentioned, and three (two of whom went down to save others) perished at the eighty fathoms, from the air all below the forty fathoms becoming so bad, as to render it imprudent, or rather impossible, for any person to go down to their assistance; and, by this time, they were unable to assist themselves. Those in this situation were drawn up by an engine, and the last who got on the rope, from the others, either, by this time, not being able to secure him properly, or not having time to do so, from him giving the signal too soon, fell from the rope, after he was within twenty fathoms of bank, and was thrown into the landing-box of the water-engine, which threw the water from the landing-box (which is situated at the fifty fathoms) to the bottom of the shaft, so that it had forty fathoms to fall. Water was also thrown from the top, with buckets, before the engine-water was diverted by this poor fellow tumbling into the landing-box. The water, by falling down the shaft, caused a circulation of air, and likewise, by absorbing the sulphurous acid gas, improved the air so much, that one who had lain at the side of the shaft, in an insensible state, for more than an hour, was restored. Other two, who were only at a very little distance from him, but were by so much further from the shaft, and consequently where the air could not be so much improved by the waterfall, were brought up immediately after him; but, though the usual modes of resuscitation were tried, neither of them could be restored. One of these last had had a very florid countenance. I took away a considerable quantity of blood from the jugular vein.

Those of a plethoric habit were much sooner, and more violently affected, than those of a spare habit, and, from what I saw, I make no doubt but one of a spare habit might remain in some degree active, whilst one of a very plethoric habit would be irrecoverably lost. When it becomes necessary for men to go into such situations, would it not be proper to take away a quantity of blood from those of a plethoric habit? I shall certainly be disposed to try it, if ever the air shall again become bad.

The candles burnt, though faintly, where the men perished, which was generally considered as an extraordinary thing; and the only hypothesis which I can conceive proper to be advanced on the subject, is, that, in a mixture of sulphurous acid gas and atmospheric air, the acrid nature of the sulphurous acid gas prevents respiration, or rather produces peculiarly deleterious effects, which, continued a certain length of time, will destroy animal life; whilst it has no further effect on combustion, than merely mechanically preventing the more free supply of oxygen it would have from pure atmospheric air, and, consequently, causing it to burn more faintly.—*Edinburgh Med. and Surg. Journal.*

---

### CRITICAL ANALYSIS

### *OF RECENT PUBLICATIONS,*

IN THE

